# Endothelial Cell Tetrahydrobiopterin Modulates Sensitivity to Ang (Angiotensin) II–Induced Vascular Remodeling, Blood Pressure, and Abdominal Aortic Aneurysm

**DOI:** 10.1161/HYPERTENSIONAHA.118.11144

**Published:** 2018-05-29

**Authors:** Surawee Chuaiphichai, Victoria S. Rashbrook, Ashley B. Hale, Lucy Trelfa, Jyoti Patel, Eileen McNeill, Craig A. Lygate, Keith M. Channon, Gillian Douglas

**Affiliations:** From the Division of Cardiovascular Medicine, British Heart Foundation Centre of Research Excellence and Wellcome Trust Centre for Human Genetics, University of Oxford, United Kingdom.

**Keywords:** angiotensin II, aorta, blood pressure, endothelial cells, vascular remodeling

## Abstract

GTPCH (GTP cyclohydrolase 1, encoded by *Gch1*) is required for the synthesis of tetrahydrobiopterin; a critical regulator of endothelial NO synthase function. We have previously shown that mice with selective loss of *Gch1* in endothelial cells have mild vascular dysfunction, but the consequences of endothelial cell tetrahydrobiopterin deficiency in vascular disease pathogenesis are unknown. We investigated the pathological consequence of Ang (angiotensin) II infusion in endothelial cell *Gch1* deficient (*Gch1*^*fl/fl*^Tie2cre) mice. Ang II (0.4 mg/kg per day, delivered by osmotic minipump) caused a significant decrease in circulating tetrahydrobiopterin levels in *Gch1*^*fl/fl*^Tie2cre mice and a significant increase in the Nω-nitro-L-arginine methyl ester inhabitable production of H_2_O_2_ in the aorta. Chronic treatment with this subpressor dose of Ang II resulted in a significant increase in blood pressure only in *Gch1*^*fl/fl*^Tie2cre mice. This finding was mirrored with acute administration of Ang II, where increased sensitivity to Ang II was observed at both pressor and subpressor doses. Chronic Ang II infusion in *Gch1*^*fl/fl*^Tie2ce mice resulted in vascular dysfunction in resistance mesenteric arteries with an enhanced constrictor and decreased dilator response and medial hypertrophy. Altered vascular remodeling was also observed in the aorta with an increase in the incidence of abdominal aortic aneurysm formation in *Gch1*^*fl/fl*^Tie2ce mice. These findings indicate a specific requirement for endothelial cell tetrahydrobiopterin in modulating the hemodynamic and structural changes induced by Ang II, through modulation of blood pressure, structural changes in resistance vessels, and aneurysm formation in the aorta.

## Abstract 2

Supplemental Digital Content is available in the text.

**See Editorial Commentary, pp 61–62**

Tetrahydrobiopterin (BH4) is a critical regulator of eNOS (endothelial NO synthase) function and eNOS-derived NO and reactive oxygen species (ROS) signaling in vascular physiology. Biosynthesis of BH4 is catalyzed by GTPCH (GTP cyclohydrolase 1, encoded by *Gch1*), the rate-limiting enzyme for de novo BH4 biosynthesis. We have previously shown that *Gch1* expression is a key determinant of BH4 bioavailability, eNOS regulation, and NO generation.^1,2^ Evidence from experimental and clinical studies have demonstrated that reduced BH4 bioavailability is associated with the pathogenesis of endothelial dysfunction, a hallmark of vascular disease states. Reduced vascular BH4 levels and eNOS uncoupling have been observed in patients with endothelial dysfunction resulting from hypertension and diabetes mellitus.^3,4^ There is increasing evidence for the role of eNOS uncoupling in the development of pathologies, such as abdominal aortic aneurysms (AAA). Gao et al^5^ demonstrated that hph-1 (hyperphenylalaninemic) mice,^6,7^ which have moderate systemic BH4 deficiency, have increased aortic ROS generation, vascular hypertrophy, and an increase in the incidence of aortic aneurysms in response to Ang (angiotensin) II. However, global BH4 deficiency in the hph-1 mouse limits the interpretation of the specific role of endothelial cell BH4 in the pathogenesis of vascular disease. We recently reported that selective deficiency in endothelial cell BH4 biosynthesis, in an endothelial cell-specific *Gch1* knockout (*Gch1*^*fl/fl*^Tie2cre) mouse, is sufficient to cause eNOS uncoupling, endothelial dysfunction, and mild hypertension, in healthy animals.^8,9^ However, it is unclear to what extent there is a specific requirement for endothelial cell BH4 in modulating susceptibility to structural vascular disease and whether selective endothelial cell BH4 deficiency is sufficient to drive pathological changes in the vascular wall.

Ang II causes vascular dysfunction, vascular smooth muscle cell (VSMC) proliferation and hypertrophy, and extracellular matrix degradation.^10^ Numerous studies have shown that Ang II–induced vascular hypertrophy and hypertension is mediated by ROS from VSMC and endothelial cells.^11–14^ Major sources of Ang II–derived ROS are the NADPH oxidases that cause eNOS uncoupling via oxidation of vascular BH4, leading to endothelial dysfunction, vascular hypertrophy, and hypertension.^15,16^ However, it is unclear whether increased endothelial cell-derived ROS from Ang II–induced eNOS uncoupling, because of selective endothelial cell BH4 deficiency, is alone sufficient to alter overall vascular function and the pathological response to Ang II stimulation, or whether these effects in endothelial cells are a consequence of disease processes throughout the vascular wall.

We sought to investigate the pathological effects of selective endothelial cell BH4 deficiency in the regulation of vascular homeostasis and blood pressure in response to Ang II, and the consequences of selective endothelial BH4 deficiency on vascular pathology, including resistance vessel remodeling and aortic aneurysm formation.

## Methods

Most of the data that support the findings of this study are available within the article and in the online-only Data Supplement. The remaining supporting data are available from the corresponding author on reasonable request.

### Conditional *Gch1* Knockout Mice

We have generated a *Gch1* conditional knockout (floxed) allele using Cre/loxP strategy as described previously.^8^ *Gch1*^*fl/fl*^ animals were bred with Tie2cre transgenic mice to produce *Gch1*^*fl/fl*^Tie2cre mice on a C57BL/6J background, where *Gch1* is deleted in endothelial cells, generating a mouse model of endothelial cell-specific BH4 deficiency. The Tie2cre transgene is active in the female germ line, so only male animals were used to establish breeding pairs to maintain conditional *Gch1* deletion. Mice were genotyped according to the published protocol.^8,17^ Male *Gch1*^*fl/fl*^Tie2cre and their *Gch1*^*fl/fl*^ littermates (wild-type [WT]) were used for all experiments at 12 to 22 weeks. All animal studies were conducted with ethical approval from the Local Ethical Review Committee and in accordance with the UK Home Office regulations (Guidance on the Operation of Animals, Scientific Procedures Act, 1986). Mice were housed in ventilated cages with a 12-hour light/dark cycle and controlled temperature (20°C–22°C) and fed normal chow and water ad libitum.

### Statistical Analysis

Data are expressed as mean±SE of the means and analyzed using GraphPad Prism version 5.0 (San Diego, CA). Comparisons between WT and *Gch1*^*fl/fl*^Tie2cre were made by unpaired Student *t* test. Comparisons with concentration-response curves were compared by 2-way ANOVA for repeated measurements. When >2 independent variables were present a 2-way ANOVA with Tukey multiple comparisons test was used. A *P* value of <0.05 was considered statistically significant.

## Results

### Modulation of GTPCH/BH4 Biosynthesis by Ang II Infusion In Vivo

To investigate whether *Gch1*/GTPCH protein and BH4 biosynthesis are modulated by Ang II–induced hypertension, we infused WT (C56BL6) mice with either saline or a pressor dose of Ang II at 1 mg/kg per day by osmotic minipump for 28 days. Ang II infusion caused a marked increase in systolic blood pressure from 7 days, which was maintained throughout 28 days of infusion (Figure S1 in the online-only Data Supplement). During the 28 days of Ang II infusion, 1 out of 8 mice died because of aortic rupture. Among the survivors, 1 out of the 7 Ang II–infused mice had an AAA at harvest compared with no AAA in the saline group (Figure S1). After 28 days of Ang II infusion, aortic BH4 and total biopterins levels were significantly increased in Ang II–infused mice compared with saline-infused mice despite no significant alteration in GTPCH protein (Figure S1). In contrast to the aorta, there was no difference in BH4 content in plasma. In addition, we found no detectable iNOS (inducible NOS) protein in aortic tissues from Ang II–infused mice (Figure S1). Interestingly, we found a significant increase in aortic DHFR (dihydrofolate reductase) protein levels in Ang II–infused mice compared with saline-infused controls, which may indicate increased recycling of BH2 to BH4.

### Ang II Infusion Exacerbates eNOS Uncoupling in Endothelial Cell BH4 Deficient Mice

**Figure 1.**
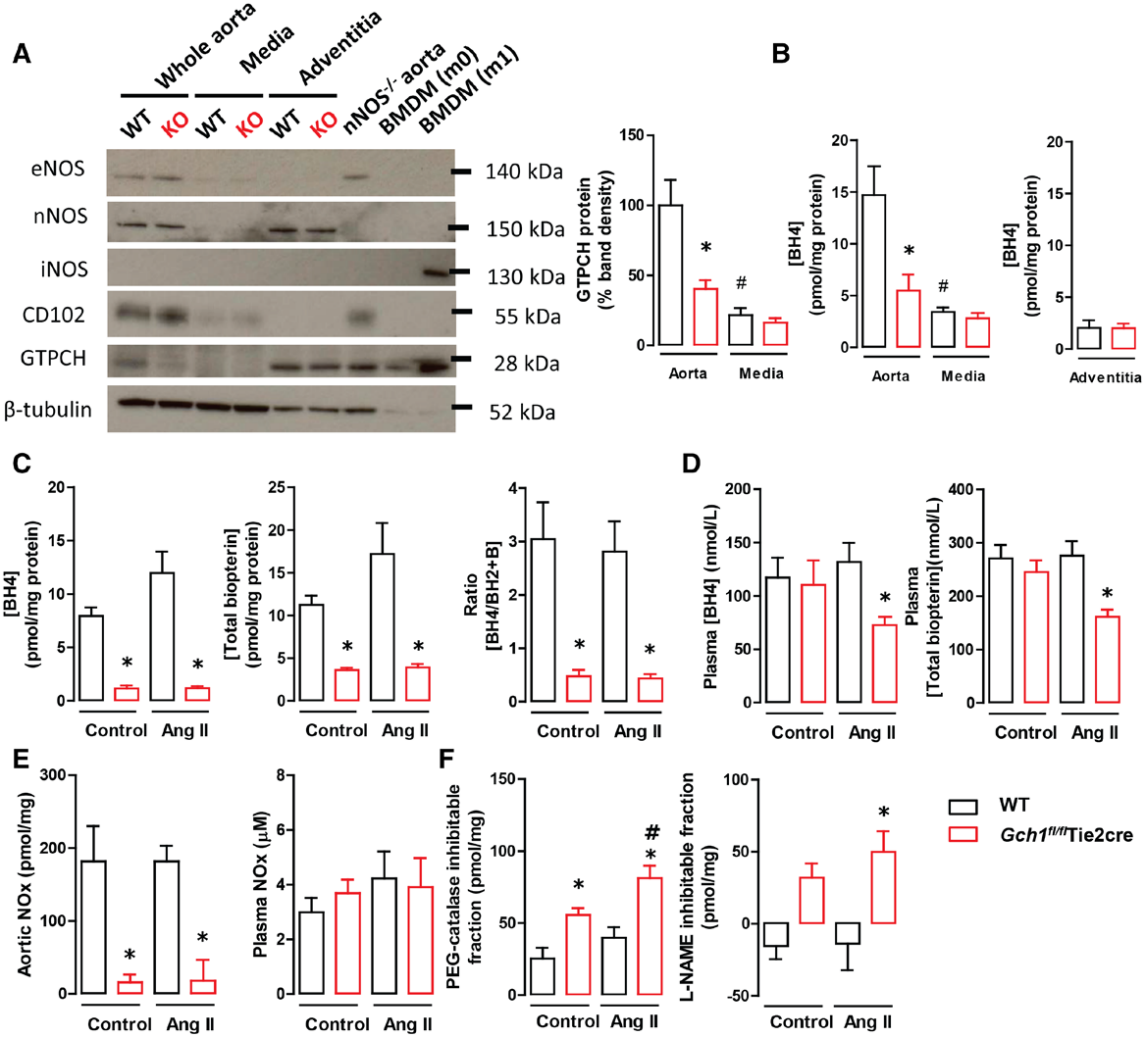
**A**, Representative immunoblots showing eNOS (endothelial NO synthase), nNOS (neuronal NOS), iNOS (inducible NOS), CD102 (endothelial cell marker), GTPCH (GTP cyclohydrolase I), and β-tubulin (loading control) proteins in whole aorta, media, and adventitia from wild-type (WT) and *Gch1*^*fl/fl*^Tie2cre knockout (KO) mice (**left**). Quantitative data, measured as percentage band density of β-tubulin, showing GTPCH protein in whole aorta and media from WT and *Gch1*^*fl/fl*^Tie2cre mice (**right**). **B**, HPLC analysis of biopterins in the whole aorta, media, and adventitia from WT and *Gch1*^*fl/fl*^Tie2cre mice (*P<0.05 comparing genotype; #*P*<0.05 comparing treatment; n=6 animals per group). **C**, Aortic tetrahydrobiopterin (BH4), total biopterins, and BH4/BH2 ratio. **D**, Plasma BH4 and total biopterins levels (*P<0.05 comparing genotype; n=5–6 animals per group). **E**, Aortic nitrite/nitrate (NOx) levels and plasma NOx levels, from WT and *Gch1*^*fl/fl*^Tie2cre mice infused with either saline or 0.4 mg/kg per day of Ang (angiotensin) II (*P<0.05 comparing genotype; n=5–6 animals per group). **F**, Aortic H_2_O_2_ production (expressed as the polyethylene glycol catalase inhibitable fraction) and aortic H_2_O_2_ production (expressed as the Nω-nitro-L-arginine methyl ester [L-NAME] inhibitable fraction) in WT and *Gch1*^*fl/fl*^Tie2cre mice infused with either saline or Ang II (*P<0.05 comparing genotype; #*P*<0.05 comparing treatment; n=6 animals per group).

After establishing the role of vascular *Gch1*/BH4 biosynthesis in hypertension in response to Ang II, we next determined whether an endothelial cell-specific reduction in BH4 plays a specific and causal role in disease pathogenesis, rather than being a consequence of the disease. We first validated the endothelial cell specificity of *Gch1* deletion in *Gch1*^*fl/fl*^Tie2cre mice. Western blot analysis showed that GTPCH protein was markedly reduced in isolated primary endothelial cells from *Gch1*^*fl/fl*^Tie2cre mice (Figure S2A). Reduced GTPCH protein was accompanied by a reduction in BH4 levels and in the BH4/BH2 ratio in endothelial cells. The decrease in BH4 resulted in an increase in the L-NAME inhibitable superoxide and ROS production at baseline (Figure S2B and S2C), indicating eNOS uncoupling in *Gch1*^*fl/fl*^Tie2cre endothelial cells. In aortas, GTPCH protein and BH4 levels were significantly reduced in *Gch1*^*fl/fl*^Tie2cre mice compared with WT mice (Figure 1A and 1B). Removal of the endothelium and adventitia (ie, generating samples of vascular media) reduced GTPCH and BH4 levels in WT aortas, such that vascular GTPCH protein and BH4 levels were no longer different between WT and *Gch1*^*fl/fl*^Tie2cre mice (Figure 1B). Similarly, BH4 levels in adventitia were similar between the genotypes (Figure 1C). This result indicates that the endothelium is the principal site for de novo BH4 biosynthesis in mouse aorta. In addition, we found that both nNOS (neuronal NOS) and eNOS proteins were present in whole aortas in both genotypes (Figure 1A). However, neither nNOS or eNOS were detected in the medial layer from either genotypes, whereas the adventitia was the predominant source of nNOS in both genotypes. In contrast to eNOS and nNOS, there was no detectable iNOS protein in either whole aorta, media, or adventitia from either genotype (Figure 1A). These results indicate that loss of BH4 from the endothelial cell layer does not impact on medial BH4 content or on the presence of NOS isoforms in the aorta.

Having demonstrated that endothelial cell BH4 deficiency leads to eNOS uncoupling in *Gch1*^*fl/fl*^Tie2cre mice, we next aimed to determine the effect of endothelial cell BH4 deficiency on the vascular response to Ang II in vivo. We infused WT and *Gch1*^*fl/fl*^Tie2cre mice with a subpressor dose (0.4 mg/kg per day) of Ang II by osmotic minipump for 5 days. In saline-infused mice, the levels of aortic BH4, total biopterins, and BH4/BH2 ratio were significantly reduced in *Gch1*^*fl/fl*^Tie2cre mice compared with WT controls (Figure 1C). In Ang II–infused mice, there was a trend toward increased vascular BH4 and total biopterin levels in WT mice, but this increase was attenuated in *Gch1*^*fl/fl*^Tie2cre mice (Figure 1C). There was no difference in circulating BH4 levels and total biopterins between groups in saline-treated mice (Figure 1D). However, Ang II infusion caused a significant reduction in circulating BH4 and total biopterin levels in *Gch1*^*fl/fl*^Tie2cre mice, whereas in WT mice circulating BH4 levels and total biopterins were unchanged after Ang II treatment, indicating that in pathological conditions, endothelial cells make a significant contribution to circulating BH4 levels. We next determined NO bioavailability in aortic tissues from Ang II–infused WT and *Gch1*^*fl/fl*^Tie2cre mice. In saline-infused mice, aortic nitrite/nitrate (NOx) accumulation was greatly decreased in *Gch1*^*fl/fl*^Tie2cre mice compared with WT control with no further reduction observed after Ang II infusion (Figure 1E). In plasma, there was no difference in circulating nitrate/nitrate between control mice and Ang II–infused mice in either WT or *Gch1*^*fl/fl*^Tie2cre mice (Figure 1E).

Because Ang II is known to increased aortic ROS generation, we next aimed to determine the impact of Ang II treatment on vascular ROS production in *Gch1*^*fl/fl*^Tie2cre mice. We observed that basal aortic H_2_O_2_ production (expressed as the polyethylene glycol catalase inhibitable fraction) was markedly increased in *Gch1*^*fl/fl*^Tie2cre mice compared with WT control and was further increased in *Gch1*^*fl/fl*^Tie2cre mice infused with Ang II. In contrast, no difference in aortic H_2_O_2_ production after Ang II infusion was observed in aortas from WT mice (Figure 1F). L-NAME markedly decreased aortic H_2_O_2_ production in *Gch1*^*fl/fl*^Tie2cre mice both at baseline and after Ang II infusion (Figure 1F), indicating that eNOS is the major source of aortic H_2_O_2_ production at baseline and after Ang II infusion. Taken together, these findings indicate that endothelial cell BH4 deficiency leads to exacerbated eNOS uncoupling and increased aortic H_2_O_2_ production in response to Ang II infusion.

### Endothelial Cell BH4 Deficient Mice Have an Increased Pressor Response to Acute Administration of Ang II

**Figure 2.**
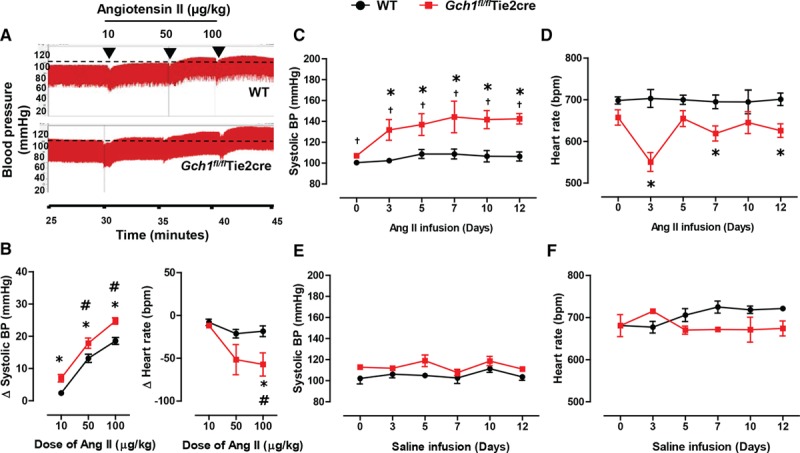
Hemodynamic response to acute and chronic Ang (angiotensin) II stimulation in *Gch1*^*fl/fl*^Tie2cre and wild-type (WT) mice. **A**, Representative continuous blood pressure (BP) traces, measured by Millar catheter, from WT and *Gch1*^*fl/fl*^Tie2cre mice before and after a serial intraperitoneal bolus doses of Ang II (10–100 μg/kg) with quantitative data for change (Δ) in **B** systolic BP and heart rate after Ang II administration. Ang II caused a significantly greater increase in systolic BP in *Gch1*^*fl/fl*^Tie2cre mice in a dose-dependent manner. Change in heart rate after Ang II administration, expressed in bpm (*P<0.05 comparing genotype; #*P*<0.05 comparing treatment; n=4–6 animals per group). **C**, Osmotic minipump containing Ang II (0.4 mg/kg per day) or saline was implanted in WT and *Gch1*^*fl/fl*^Tie2cre mice. Systolic BP and heart rate, measured by noninvasive tail cuff, in WT and *Gch1*^*fl/fl*^Tie2cre mice during 14 d of Ang II infusion. Systolic BP significantly increased in *Gch1*^*fl/fl*^Tie2cre mice after 3 d of Ang II infusion but was unaltered in WT mice (*P<0.05 comparing treatment; †*P*<0.05 comparing genotype; n=5–6 animals per group). **D**, Heart rate significantly reduced in *Gch1*^*fl/fl*^Tie2cre mice after Ang II infusion but unchanged in WT mice. **E** and **F**, Systolic BP and heart rate respectively in saline-infused WT and *Gch1*^*fl/fl*^Tie2cre mice (n=3 animals per group).

To determine the functional importance of increased ROS production by eNOS in *Gch1*^*fl/fl*^Tie2cre mice, we measured the hemodynamic response to acute Ang II infusion. Using Millar catheters to measure invasive blood pressure changes, acute Ang II administration at a subpressor dose of 10 μg/kg, caused a significant increase in systolic blood pressure only in *Gch1*^*fl/fl*^Tie2cre mice (change from baseline, 7.0±1.2 versus 2.4±0.4 mm Hg; *P<0.05*). At pressor doses of Ang II, blood pressure was significantly augmented at both 50 and 100 μg/kg in *Gch1*^*fl/fl*^Tie2cre mice compared with WT controls (Figure 2A and 2B). Heart rate was markedly decreased in response to Ang II in both WT and *Gch1*^*fl/fl*^Tie2cre mice at both the 50 and 100 μg/kg dose of Ang II. However, a greater reduction in heart rate was observed in *Gch1*^*fl/fl*^Tie2cre mice compared with WT littermates (Figure 2B). These findings suggest that endothelial cell BH4 deficiency confers increased sensitivity to acute Ang II.

### Increased Arterial Blood Pressure in Response to Chronic Ang II Infusion in Endothelial Cell BH4 Deficient Mice

We next aimed to establish whether the acute increase in blood pressure observed in *Gch1*^*fl/fl*^Tie2cre mice persisted during chronic Ang II infusion. Mice were infused with Ang II at a subpressor dose (0.4 mg/kg per day) for 14 days and blood pressure measured in conscious mice by the tail cuff. As expected, this subpressor dose of Ang II infusion did not have any effect on the systolic blood pressure of WT mice at any time point studied (Figure 2C). In contrast, the small increase in basal systolic blood pressure in *Gch1*^*fl/fl*^Tie2cre mice (108±1 versus 101±2 mm Hg; *P<*0.05;Figure 2C) was greatly increased in *Gch1*^*fl/fl*^Tie2cre mice as soon as 3 days after low-dose Ang II infusion (132±10 versus 102±2 mm Hg; *P<*0.05) and was maintained throughout the 14 day infusion. Basal heart rate was significantly reduced in *Gch1*^*fl/fl*^Tie2cre mice compared with WT littermates from day 3 onwards of Ang II infusion but remained unchanged in WT mice (551±23 versus 703±21 bpm; *P<*0.05; Figure 2D). Saline infusion had no significant effect on either systolic blood pressure or heart rates in either WT or *Gch1*^*fl/fl*^Tie2cre mice (Figure 2E and 2F).

### Chronic Ang II Infusion Exacerbates Vascular Dysfunction in Resistance Mesenteric Arteries in *Gch1*^*fl/fl*^Tie2cre Mice

**Figure 3.**
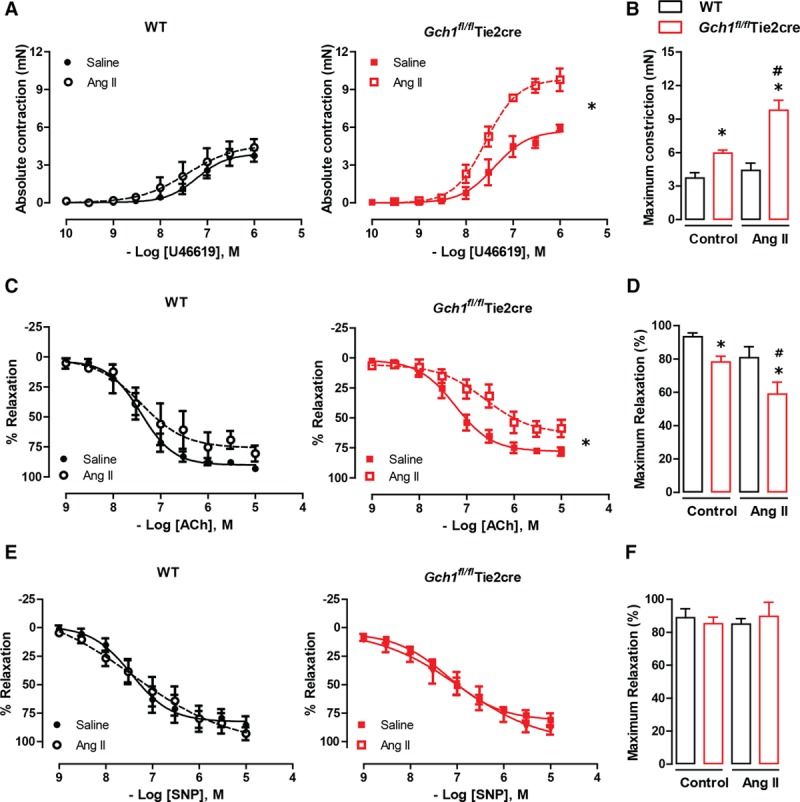
Impaired vasoconstrictions and endothelium-dependent vasodilatations in *Gch1*^*fl/fl*^Tie2cre resistance mesenteric arteries in response to Ang (angiotensin) II stimulation. Isometric tension study of second-order resistance mesenteric arteries from wild-type (WT) and *Gch1*^*fl/fl*^Tie2cre mice with either Ang II or saline infusion for 28 d. **A**, Vasoconstrictions in response to vasoconstrictor U46619 in mesenteric arteries. **B**, Maximum contraction (mN) in response to 1 µmol/L U46619. **C**, Endothelium-dependent vasodilatations in response to acetylcholine (ACh) in mesenteric arteries. **D**, Maximum relaxation (%) in response to 10 µmol/L ACh. **E**, Endothelium-independent vasodilatations in response to sodium nitroprusside (SNP) in mesenteric arteries. **F**, Maximum relaxation (%) in response to 10 µmol/L SNP (*P<0.05 comparing genotype; #*P*<0.05 comparing treatment; n=4–6 animals per group).

We next investigated the effect of eNOS uncoupling on vasomotor function in response to chronic Ang II infusion in isolated second-order resistance mesenteric arteries. In saline-treated mice, vasoconstrictions to the thromboxane A_2_ receptor mimetic, U46619, were significantly enhanced in *Gch1*^*fl/fl*^Tie2cre mesenteric arteries compared with WT controls. Ang II infusion (0.4 mg/kg per day for 28 days) caused a further increase in vasoconstrictions to U46619 in mesenteric arteries from *Gch1*^*fl/fl*^Tie2cre mice compared with saline-treated *Gch1*^*fl/fl*^Tie2cre mice (Figure 3A). In contrast, Ang II infusion had no significant effect on vasoconstriction to U46619 in WT mice. A significant increase in maximum vasoconstrictions to U46619 (1 µmol/L) was observed at baseline in saline-treated *Gch1*^*fl/fl*^Tie2cre mice and was further augmented after Ang II infusion (5.9±0.3 versus 9.7±0.9 mN; *P*<0.05) with no difference observed in WT mice infused with Ang II (3.7±0.4 versus 4.4±0.7 mN; Figure 3B).

In saline-treated mice, a significant blunting of endothelial-dependent vasodilation was observed in mesenteric arteries from *Gc11*^*fl/fl*^Tie2cre mice (Figure 3C). This difference was even more pronounced after Ang II infusion, with a further impairment in endothelial-dependent relaxation observed in *Gch1*^*fl/fl*^Tie2cre mice. This was highlighted by the significant decrease in the maximal dilation response observed in mesenteric arteries from *Gch1*^*fl/fl*^Tie2cre mice after Ang II infusion (78.1±3.4% versus 58.9±7.2%; *P*<0.05; Figure 3D). In contrast, Ang II had no significant effect on endothelium-dependent vasodilatation in WT mice, with no significant difference observed in maximum relaxation after Ang II treatment (93.3±2.2% versus 80.7±6.6%; Figure 3C and 3D).

Furthermore, there were no difference in endothelium-independent vasodilatation between WT and *Gch1*^*fl/fl*^Tie2cre mice in either the saline or Ang II group, suggesting the impairment of vasodilatation to acetylcholine observed in *Gch1*^*fl/fl*^Tie2cre mesenteric arteries was not because of an alteration in vascular smooth muscle sensitivity to exogenous NO (Figure 3E and 3F).

### eNOS Uncoupling Leads to Vascular Remodeling in *Gch1*^*fl/fl*^Tie2cre Resistance Mesenteric Arteries in Response to Ang II Infusion

**Figure 4.**
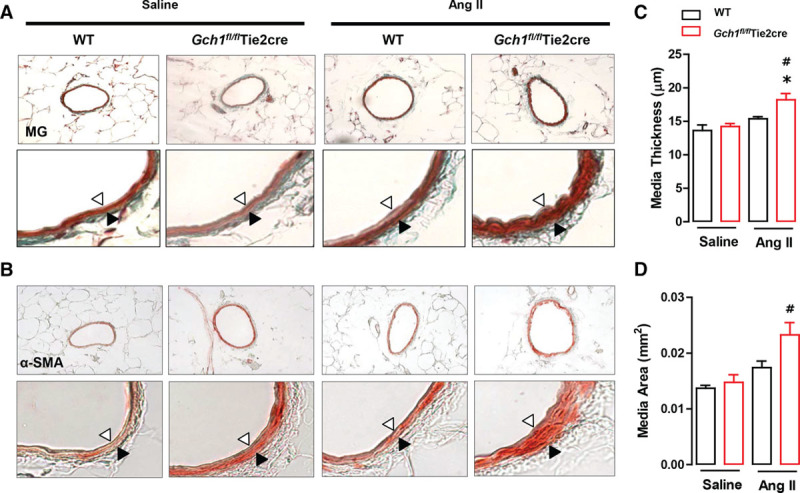
Exacerbated resistance mesenteric artery remodeling in *Gch1*^*fl/fl*^Tie2cre mice in response to Ang (angiotensin) II infusion. Vascular remodeling was analyzed in embed sections of the second-order branch of mesenteric arteries (perfusion fixed at 100 mm Hg) from wild-type (WT) and *Gch1*^*fl/fl*^Tie2cre mice with either Ang II or saline infusion (0.4 mg/kg/ per day, 28 d). Representative images are shown (**A**) Masson–Goldner (MG) staining and (**B**) α-smooth muscle actin (α-SMA) staining of mesenteric arteries. Vascular remodeling was evaluated by (**C**) media thickness and (**D**) media area. Opened arrow indicates internal elastic lamina and closed arrow indicates external elastic lamina. (*P<0.05 comparing genotype; #*P*<0.05 comparing treatment; n=4–6 animals per group).

Given the marked difference in constrictor response between the 2 groups after Ang II treatment and the role of vascular ROS production in vascular remodeling and hypertrophy, we next assessed medial changes in resistance arteries. Second-order mesenteric arteries from *Gch1*^*fl/fl*^Tie2cre mice and WT mice were perfusion fixed at 100 mm Hg after 28 days of saline or Ang II infusion. Ang II caused a marked increase in medial thickness and medial area in mesenteric arteries from *Gch1*^*fl/fl*^Tie2cre mice (Figure 4A through 4D). In contrast, no difference in either medial area of thickness was observed in mesenteric arteries from WT mice. In saline-infused mice, there was no significant difference in medial thickness and medial area between mesenteric arteries of WT and *Gch1*^*fl/fl*^Tie2cre mice (Figure 4A through 4D). Taken together, these results suggest that selective deficiency in endothelial cell BH4 biosynthesis leads to vascular dysfunction and medial remodeling in resistance arteries in response to chronic Ang II infusion.

### Increased AAA Formation in Endothelial Cell BH4 Deficient Mice After Ang II Infusion

**Figure 5.**
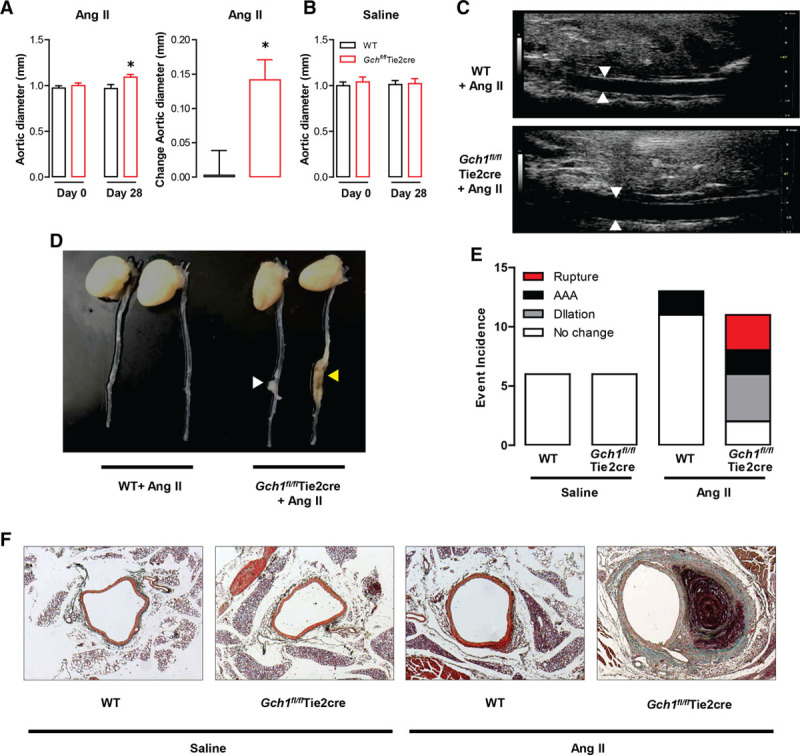
Ang (angiotensin) II–induced vascular pathology of abdominal aortic aneurysm (AAA) in *Gch1*^*fl/fl*^Tie2cre mice. Wild-type (WT) and *Gch1*^*fl/fl*^Tie2cre mice were treated with a subpressor dose (0.4 mg/kg per day) of Ang II by osmotic minipump for 28 d. The lumen diameter of abdominal aortas was assessed in mice before and at the end of 28 d of Ang II infusion by ultrasound. **A**, In Ang II–infused mice, there was no significant difference in the diameter of abdominal aortas between WT and *Gch1*^*fl/fl*^Tie2cre mice at day 0. After 28 d Ang II infusion, the lumen diameter of abdominal aorta of *Gch1*^*fl/fl*^Tie2cre mice was significantly increased compared with WT controls. Ang II infusion caused a greater increase in aortic lumen diameter (expressed as changed in lumen diameter) in *Gch1*^*fl/fl*^Tie2cre mice compared with WT mice (*P<0.05 comparing genotype; n=8–9 per group). **B**, In saline-infused mice, there was no significantly difference in the lumen diameter of abdominal aortas between WT and *Gch1*^*fl/fl*^Tie2cre mice either at day 0 or day 28 of saline infusion (n=5–6 per group). **C**, Representative of aortic lumen diameter by ultrasound after Ang II infusion in WT and *Gch1*^*fl/fl*^Tie2cre mice. **D**, Representative appearance of aortic dilation (opened arrow) and abdominal aortic aneurysm formation (yellow arrow). **E**, Event incidence of AAA pathology: dilation without AAA, AAA formation, and AAA rupture with sudden death in Ang II–infused *Gch1*^*fl/fl*^Tie2cre and WT mice. **F**, Representative images of Masson–Goldner staining of abdominal aortic sections from WT and *Gch1*^*fl/fl*^Tie2cre mice after saline or Ang II infusion for 28 d.

Having demonstrated increased vascular ROS and increased sensitivity in blood pressure and resistance artery function in response to Ang II, we next aimed to establish the consequence of endothelial cell BH4 deficiency on conduit artery remodeling. We infused WT and *Gch1*^*fl/fl*^Tie2cre mice with a subpressor dose (0.4 mg/kg per day) of Ang II by osmotic minipump for 28 days. The lumen diameter of abdominal aortas was assessed in mice before and at the end of 28 days of Ang II infusion, using ultrasound. There was no significant difference in the diameter of the abdominal aortas between WT and *Gch1*^*fl/fl*^Tie2cre mice at baseline (day 0; Figure 5A). However, after 28 days of Ang II infusion, the lumen diameter of the abdominal aorta in *Gch1*^*fl/fl*^Tie2cre mice was significantly increased compared with WT controls (Figure 5A and 5C). In saline-infused mice, there was no significant difference in the lumen diameter of the abdominal aortas between WT and *Gch1*^*fl/fl*^Tie2cre mice either at day 0 or day 28 (Figure 5B).

During 28 days of Ang II infusion, 3 out of 11 *Gch1*^*fl/fl*^Tie2cre mice died because of aortic rupture. In contrast, no WT mice died during the study period. Among the survivors, 75% (6 out of 8) of Ang II–infused *Gch1*^*fl/fl*^Tie2cre mice had classical features of AAA: aortic dilatation (50%; 4 out of 8 mice) and AAA formation (25%; 2 out of 8 mice; Figure 5D and 5E). In contrast, only 2 out of 13 WT mice showed the pathology of AAA formation. Importantly, none of the saline-infused WT or *Gch1*^*fl/fl*^Tie2cre mice died or developed any features of AAA pathology (Figure 5E). Histological sections of suprarenal aortas stained with Masson–Goldner demonstrated aortic dilatation and AAA formation in the suprarenal aorta after 28 days of Ang II infusion in *Gch1*^*fl/fl*^Tie2cre mice (Figure 5F).

As Tie2cre can lead to cre-mediated gene deletion in hematopoietic cells, we next assessed whether Ang II stimulation affects GTPCH/BH4 biosynthesis in macrophages. As expected, at baseline GTPCH protein was barely detectable in macrophages from *Gch1*^*fl/fl*^Tie2cre mice compared with WT controls (Figure S3A and S3B). This was accompanied by a marked reduction in BH4 and total biopterins in *Gch1*^*fl/fl*^Tie2cre macrophages compared with WT controls (Figure S3C). In addition, iNOS protein was undetectable in either WT or *Gch1*^*fl/fl*^Tie2cre macrophages. Ang II stimulation at 1 μmol\L for 16 hours did not alter GTPCH protein, BH4, or total biopterins levels in either WT or *Gch1*^*fl/fl*^Tie2cre macrophages compared with the control untreated group. Unlike lipopolysaccharide and IFNγ (interferon γ) stimulation, Ang II did not cause induction of iNOS protein either in WT or *Gch1*^*fl/fl*^Tie2cre macrophages.

## Discussion

In this study, we used a mouse model of endothelial cell-targeted *Gch1* deletion to test the requirement for endothelial cell BH4 in modulating the pathological vascular changes associated with Ang II stimulation. The major findings of this study are (1) loss of endothelial cell BH4 is sufficient to increase the sensitivity to Ang II–mediated hemodynamic responses; (2) Ang II infusion exacerbates eNOS uncoupling, increasing aortic eNOS-derived H_2_O_2_ production in *Gch1*^*fl/fl*^Tie2cre mice; (3) endothelial cell-specific *Gch1* deficiency causes medial hypertrophy and dysfunction in resistance arteries in response to chronic Ang II infusion; and (4) chronic Ang II infusion leads to pathological vascular remodeling with a greatly increased incidence of AAA in *Gch1*^*fl/fl*^Tie2cre mice. Collectively, our studies demonstrate a specific role for endothelial cell BH4 in the pathogenesis of Ang II–mediated vascular hypertrophy and dysfunction, hypertension, and the development of AAA.

It is well-established that Ang II promotes hypertension through increased oxidative stress, vascular remodeling, and endothelial dysfunction.^18,19^ Increased levels of Ang II and angiotensin-converting enzyme, because of the DD genotype, are associated with hypertension and increased cardiovascular disease, respectively.^20^ Recently, the important role for vascular ROS signaling in the development of vascular remodeling and hypertension has been demonstrated in mice deficient in eNOS and NADPH oxidase.^21,22^ In our current study, we have also shown for the first time that endothelial cell BH4 deficiency and the associated changes in ROS and eNOS coupling are alone sufficient to increase the sensitivity of the acute pressor response to Ang II. We have shown that even low doses of Ang II, that are insufficient to increase blood pressure in wild-type mice, cause pressor responses in *Gch1*^*fl/fl*^Tie2cre mice. Furthermore, this increased sensitivity to Ang II, conferred by endothelial cell-specific BH4 deficiency, is sufficient to induce both functional and structural abnormalities in both resistance and conduit arteries.

To assess the physiological importance of endothelial cell BH4, we utilized a chronic model of Ang II infusion. It has been reported that chronic administration of a subpressor dose of Ang II mimics the development of human hypertension to a greater extent than the pressor dose.^23^ In this study, we, therefore, used the subpressor dose of Ang II (0.4 mg/kg per day) to investigate the pathological role of reduced endothelial cell *Gch1* expression in the development of Ang II–induced hypertension. We found that a chronic subpressor infusion of Ang II resulted in a significant increase in blood pressure only in *Gch1*^*fl/fl*^Tie2cre mice. A growing body of evidence has suggested that ROS and NO play a critical role in the regulation of AT1R (angiotensin 1 receptor) signaling. Indeed, endogenous ROS increases AT1R density in rat cardiac fibroblasts,^24^ whereas NO decreases Ang II signaling by downregulating AT1R density through S-nitrosylation in the heart.^24,25^ It is possible that the alteration in NO/ROS signaling in *Gch1*^*fl/fl*^Tie2cre mice leads to increased Ang II sensitivity because of alteration in receptor density. In addition, it is possible that the increased baseline arterial ROS production observed in *Gch1*^*fl/fl*^Tie2cre mice primes the vasculature for an enhanced Ang II response. Indeed, it has been reported that vascular Nox expression, activity, and the associated ROS generation are increased in resistance arteries from human hypertensives, which is associated with increased sensitivity to Ang II stimulation.^26,27^ The results of this study indicate a critical role of endothelial cell BH4 in the sensitivity, and hence the resultant hemodynamic response to Ang II.

Hypertension has been shown to be associated with the impaired vascular function, particularly in resistance arteries, the key site for arterial blood pressure regulation. Previous studies have demonstrated eNOS uncoupling in resistance vessels from DOCA (deoxycorticosterone acetate)-salt and Ang II–induced hypertensive mice.^16,28^ However, what is unclear from these studies is whether eNOS uncoupling is causative in the resulting vascular dysfunction or was part of the disease process. In our study, we show for the first time that endothelial cell BH4 deficiency is alone sufficient to cause Ang II–mediated vascular dysfunction. We observed a further enhancement of the vasoconstrictor response and significantly impaired endothelium-dependent vasodilatation in resistance mesenteric arteries from Ang II–infused *Gch1*^*fl/fl*^Tie2cre mice. This is in contrast to wild-type mice, where no difference in the constrictor or dilator responses were observed with Ang II infusion. Previous studies have demonstrated that increased level of superoxide and H_2_O_2_ enhanced Ang II–stimulated redox signaling in resistance arteries from hypertensive patients.^26^ The combination of increased eNOS-derived ROS and reduced eNOS-derived NO production is likely to be the major contributor toward the development of vascular dysfunction, and hence hypertension observed in Ang II–treated *Gch1*^*fl/fl*^Tie2cre mice and contrasts with eNOS knockout mice where all functions of eNOS (ie, both NO and ROS generation) are deleted.

It has been demonstrated that vascular H_2_O_2_ plays a crucial role in smooth muscle growth and hypertrophy. Chronic Ang II infusion increases vascular superoxide and H_2_O_2_-derived NADPH oxidase, subsequently leading to vascular remodeling.^12–14^ In addition, treatment with either an Ang II receptor antagonist or an angiotensin-converting enzyme inhibitor reduced ROS generation and vascular hypertrophy in spontaneously hypertensive rats.^29^ Our studies now highlight the role for increased ROS generation specifically from endothelial cells, because of eNOS uncoupling, in the pathogenesis of Ang II–induced vascular remodeling. Ang II infusion at a subpressor dose caused a marked increase in mesenteric resistance artery medial thickness and area (typical feature of hypertrophic remodeling) in *Gch1*^*fl/fl*^Tie2cre mice, but no change in WT controls. It is possible that the increased medial area was in part because of increased pressor response to Ang II. However, it is unlikely that the increased blood pressure alone was responsible for the medial remodeling. Several in vivo studies have demonstrated that noradrenaline infusion, at concentrations producing a similar degree of hypertension to Ang II infusion, does not cause vascular hypertrophy.^30^ In endothelial cell-specific BH4 deficient mice, it is likely that increased endothelial cell ROS/H_2_O_2_ production, because of eNOS uncoupling, activates and primes the underlying VSMC, which in turn promote vascular hypertrophy in response to Ang II. Consistent with this notion, administration of polyethylene glycol catalase or overexpression of catalase in VSMC has been reported to reduce vascular H_2_O_2_ production and subsequently vascular hypertrophy in response to chronic Ang II stimulation.^31^

Important roles for eNOS uncoupling and BH4 deficiency in AAA formation have been recently reported in hph-1 mice, which have a moderate systemic BH4 deficiency because of a global reduction in *Gch1* expression. Hph-1 mice treated with Ang II at 0.7 mg/kg per day, show an increased eNOS uncoupling and AAA formation.^5^ However, the hph-1 mouse has a global BH4 deficiency, affecting all cells and tissues, so is not possible to dissect out how BH4 deficiency mediates the increase in vascular pathology. Notably, in the present studies, Ang II infusion even at a subpressor dose (0.4 mg/kg per day) greatly increased the incidence of AAA in *Gch1*^*fl/fl*^Tie2cre mice, associated with eNOS uncoupling and increased aortic eNOS-derived H_2_O_2_ generation. Vascular H_2_O_2_ has been previously implicated in the pathology of AAA in various mouse models of AAA, including the Ang II, elastase, and CaCl_2_ model.^16,32,33^ Indeed, H_2_O_2_ has been reported to promote MMP-3 (matrix metalloproteinase-3), MMP-9, and MMP-12 expression, which are implicated in extracellular matrix degradation that is important in the pathogenesis of AAA.^34^ Administration of polyethylene glycol catalase or overexpression of catalase in VSMC has been reported to reduce the formation of AAA by enhancing VSMC survival and decreasing MMP activity.^33^ We have previously shown that increased endothelial cell superoxide by Nox-2 overexpression is sufficient to predispose mice to AAA formation.^22^ Taken together, these data strongly indicate that increased endothelial cell H_2_O_2_ production because of eNOS uncoupling is sufficient to increase AAA formation.

Ang II can promote inflammatory cell recruitment into the vascular wall and in particular to the abdominal aorta region.^35^ In the present study, we observed no difference in aortic protein levels of either iNOS or GTPCH between Ang II–treated mice and the saline controls after 28 days of Ang II infusion. These findings suggest that Ang II is unlikely to play a role in modulating aortic *Gch1*/BH4 biosynthesis or iNOS protein levels. In addition, Ang II stimulation of bone marrow-derived macrophages did not alter BH4 levels, GTPCH protein, or iNOS protein expression. Hence, the lack of *Gch1* in myeloid cells is unlikely to be the cause of the increase in AAA formation in *Gch1*^*fl/fl*^Tie2cre mice; however, additional mechanistic studies would be required to confirm this.

Despite an increased aortic eNOS-derived H_2_O_2_ production in *Gch1*^*fl/fl*^Tie2ce mice, the levels of BH4, total biopterins, and BH4/BH2 ratio were unaffected by Ang II stimulation in *Gch1*^*fl/fl*^Tie2cre aortas. It is likely that other mechanisms, independent of endothelial cell BH4 levels, may contribute to further exacerbate eNOS uncoupling in response to Ang II stimulation. Indeed, several reports have demonstrated that Ang II can lead to eNOS uncoupling by eNOS-glutathionylation, independent of BH4 levels, in several experimental models of hypertension.^36,37^ In addition, it is possible that the increased blood pressure in response to Ang II may contribute to the development of AAA pathology in *Gch1*^*fl/fl*^Tie2cre mice. However, increased blood pressure is unlikely to be the cause of AAA formation in response to Ang II infusion^5,22^ because numerous studies have shown that blood pressure matching with noradrenaline does not induce AAA formation.^22,38^

A previous study reported that Ang II infusion at 0.7 mg/kg per day for 14 days caused a decrease in aortic BH4 levels because of a reduction in endothelial cell DHFR protein.^5^ However, consistent with our findings, Kossmann et al^39^ demonstrated that Ang II infusion at 1 mg/kg per day for 7 days increased aortic GTPCH protein and BH4 levels. The authors hypothesized that this increase was because of increased inflammatory cell influx as iNOS protein was detectable in the vascular wall after Ang II infusion. In contrast, we found no detectable iNOS protein in aortic tissues from Ang II-infused mice, suggesting that at the later time point inflammatory cells are either no longer abundant or are not expressing iNOS. Interestingly, we found a significant increase in aortic DHFR protein levels in Ang II–infused mice compared with saline-infused controls. This finding indicates that the increase in aortic BH4 and total biopterin content may be because of increased DHFR-mediated recycling of BH2 to BH4, rather than the presence of proinflammatory inflammatory cells. The increase in aortic BH4 in response to Ang II may represent a vascular protective mechanism. This result is consistent with the finding of an enhanced pathological response to Ang II in *Gch1*^*fl/fl*^Tie2cre mice, which are unable to upregulate aortic BH4 content in response to Ang II by either the de novo or salvage pathway. This increased aortic BH4 content may act as a protective measurement to compensate for the increase vascular oxidative stress in response to Ang II.

Interestingly, Ang II had no significant effect on plasma BH4 levels in WT mice, whereas plasma BH4 levels were greatly decreased in Ang II–infused *Gch1*^*fl/fl*^Tie2cre mice, indicating that under these pathological conditions endothelial cells make a significant contribution to circulating BH4 levels. Thus, circulating BH4 levels may be a useful indicator of AAA. Consistent with this finding, Gao et al^5^ demonstrated that decreased circulating BH4 is correlated with reduced vascular BH4 levels, which is associated with AAA formation in hph-1 mice. It would be interesting to determine if supplementation with BH4 could reverse the pathological changes observed in the current study. However, it has been challenging to achieve arterial BH4 supplementation because of the highly oxidative nature of BH4. Hence, alternative treatment strategies such as 5-methyl-tetrahydrofolate or folate, which have been shown to increase vascular BH4 levels in patients undergoing coronary artery bypass grafts, may represent a viable alternative.^40^

In summary, we describe for the first time a specific requirement for endothelial cell BH4 in modulating the hemodynamic and structural changes induced by Ang II, through modulation of blood pressure, structural changes in resistance vessels, and aneurysm formation in the aorta. This study demonstrates a specific role for endothelial cell BH4 deficiency/eNOS uncoupling in the pathogenesis of Ang II driven vascular disease.

## Perspectives

Selective targeting endothelial cell *Gch1* and BH4 biosynthesis under pathological conditions may provide a novel therapeutic target for the treatment of vascular disease.

## Sources of Funding

This study was supported by a British Heart Foundation Program Grant (RG/12/5/29576), Chair award (CH/16/1/32013), Oxford British Heart Foundation Centre of Research Excellence (RE/13/1/30181), Wellcome Trust (090532/Z/09/Z), and the National Institute for Health Research Oxford Biomedical Research Centre.

## Disclosures

None.

## Supplementary Material

## Novelty and Significance

### What Is New?

- This is the first study to show that endothelial cell tetrahydrobiopterin deficiency is alone sufficient to cause pathophysiological changes in large and small vessels in response to the pathophysiological stimulus Ang (angiotensin) II.

### What Is Relevant?

- Targeting endothelial cell *Gch1* and tetrahydrobiopterin biosynthesis under pathological conditions may provide a novel therapeutic target for the treatment of Ang II driven vascular disease.

### Summary

Endothelial NO synthase uncoupling, because of selective deficiency in endothelial cell tetrahydrobiopterin biosynthesis, leads to vascular remodeling and vascular dysfunction in resistance vessels, hypertension, and an increased incidence of aneurysm formation in response to a subpressor does of Ang II. These findings indicate a specific requirement for endothelial cell tetrahydrobiopterin in modulating the hemodynamic and structural changes induced by Ang II, through modulation of blood pressure, structural changes in resistance vessels, and aneurysm formation in the aorta.
