# Supplementary material for: Endothelial Cell Tetrahydrobiopterin Modulates Sensitivity to Ang (Angiotensin) II–Induced Vascular Remodeling, Blood Pressure, and Abdominal Aortic Aneurysm
Source: Hypertension. 2018 May 29;72(1):128–38. doi: 10.1161/HYPERTENSIONAHA.118.11144 (PMC6012043; doi:10.1161/HYPERTENSIONAHA.118.11144)
Supplement: Supplementary file 1 [file hyp-72-128-s001.pdf]

SUPPLEMENTAL MATERIAL for

Endothelial Cell BH4 Modulates Sensitivity to Angiotensin II-Induced Vascular Remodelling, Blood Pressure and AAA

Surawee Chuaiphichai, Victoria S. Rashbrook, Ashley B. Hale, Lucy Trelfa, Jyoti Patel, Eileen McNeill, Craig A. Lygate, Keith M. Channon*, Gillian Douglas

British Heart Foundation Centre of Research Excellence,

Division of Cardiovascular Medicine, University of Oxford

Wellcome Trust Centre for Human Genetics, University of Oxford

***Corresponding Author:**

Professor Keith M. Channon,
Division of Cardiovascular Medicine,
Radcliffe Department of Medicine,
University of Oxford,
John Radcliffe Hospital,
Oxford, OX3 9DU, UK
Tel: +44-1865-572783
Email: keith.channon@cardiov.ox.ac.uk

SUPPLEMENTAL METHODS

Tissue Collection

Tissue for histological analysis was collected from mice perfused with phosphate buffer saline (PBS) followed by 4% paraformaldehyde. Tissue for biochemical analysis was collected from mice perfused with PBS only. Plasma was collected from heparinised blood samples spun at 3000g for 10 min at 4 °C and stored at -80°C until analysis. Tissue for biochemical analysis was snap frozen in liquid nitrogen and stored at -80°C until analysis. Primary endothelial cells were isolated from lungs by immunoselection with CD31 antibody (BD Biosciences, UK) coated magnetic beads as described previously.[1] Bone marrow was obtained by flushing the femur and tibia of adult mice with PBS. A single cell suspension was prepared by passing the bone marrow through a 70 mm cell strainer. Cells were cultured in 10 cm non tissue culture treated dishes for 7 days in DMEM:F12 (Invitrogen) supplemented with 100 U/ml penicillin and 100 ng/ml streptomycin (Sigma), 10% (v/v) fetal bovine serum (PAA Laboratories), 5 mM L-glutamine (Sigma), and 10–15% (v/v) L929 conditioned medium. Following differentiation, cells were harvested and plated into 6-well plates containing serum-free media (Optimem supplemented with 100 U/ml penicillin and 100 ng/ml streptomycin and 0.2% (w/v) low-endotoxin bovine serum albumin (Sigma)). Cells were stimulated with 1 µM Ang II (Sigma) for 16 h; parallel wells were left unstimulated. After 16 h cell pellets were collected and the cells subjected to biochemical analysis.

Determination of Tissue Tetrahydrobiopterin Levels

BH4 and oxidised biopterins (BH2 and biopterin) were determined by high-performance liquid chromatography (HPLC) followed by electrochemical and fluorescence detection, respectively, following an established protocol.[2] Briefly, tissues arteries and aortas and cells were freeze-thawed in ice-cold resuspension buffer (50 mmol·L^{-1} phosphate-buffered saline, 1 mmol·L^{-1} dithioerythriol, 1 mmol·L^{-1} EDTA, pH 7.4). After centrifugation at 13,200 rpm for 10 min at 4°C, supernatant was removed and ice-cold acid precipitation buffer (1 mol·L^{-1} phosphoric acid, 2 mol·L^{-1} trichloroacetic acid, 1 mmol·L^{-1} dithioerythritol) was added. Following centrifugation at 13,200 rpm for 10 min at 4°C, the supernatant was removed and injected onto the HPLC system. Quantification of BH4 and oxidised biopterins was obtained by comparison with external standards and normalised to protein concentration, determined by the bicinchoninic acid protein assay.

Quantification of Superoxide Production

Superoxide production from primary endothelial cells from 12-22 week old mice was measured by quantifying the accumulation of 2-hydroxyethidium and ethidium by HPLC as previously described.[3] Endothelial cells were extracted as above and superoxide production assayed from cells straight off the column. Cells were incubated at 37 °C in Krebs-HEPES buffer with or without PEG-SOD (100U/ml) for 20 min. Cells were then treated with dihydroethidium (25 µM; DHE) 20 minutes. Separation of dihydroethidium, 2-hydroxyethidium and ethidium was performed using a gradient HPLC system (Jasco, UK) with an ODS3 reverse phase column (250 mm, 4.5 mm;

Hichrom, UK), and quantified using a fluorescence detector set at 510 nm (excitation) and 595 nm (emission).

Insertion of Osmotic Mini-Pumps

Male *Gch1*$^{fl/fl}$Tie2cre and wild-type mice (16-22 week-old mice) were anaesthetised by inhalation of 2% isofluorane in 100% oxygen. Osmotic mini-pumps were implanted subcutaneous (Alzet Model 1002, 14 days: Charles River) in the mid-scapular region. Mini-pumps delivered either saline or 0.4 mg/kg/day angiotensin II (Ang II; Sigma-Aldrich, Gillingham, UK).

Non-Invasive Blood Pressure Measurement Using Tail-Cuff

Blood pressure in conscious wild-type and *Gch1*$^{fl/fl}$Tie2cre mice was measured using the VisitechR tail-cuff plethysmography system. Experiments were performed between the hours of 8:00 am and 12.00 pm. Twenty readings were taken per mouse of which the first 5 readings were discarded. The remaining 15 readings were used to calculate the mean systolic blood pressure and heart rate in each mouse. Following a 5 day training period and 3 days baseline recordings, osmotic minipumps filled with either angiotensin II (0.4 mg/kg/day) or saline (PBS) was implanted in wild-type and *Gch1*$^{fl/fl}$Tie2cre mice. Blood pressure recordings were then obtained on day 3, 5, 7 10 and 12 post-implantation.

Invasive Blood Pressure Measurement Using Millar Catheter

Systolic blood pressure and heart rate were measured in wild-type and *Gch1*$^{fl/fl}$Tie2cre mice by a direct invasive approach under terminal anaesthesia using a Millar catheter. The left carotid artery was isolated via a midline incision in the neck and a 1.4F Millar catheter was introduced into the aorta via the carotid artery. Experimental anaesthesia was monitored to maintain a respiratory rate of 60 to 70 breaths per minutes, mice were warmed to maintain a body temperature of 37 °C. Following a 15-minute equilibration period, blood pressure data was collected continuously for at least 15 minutes (as baseline) and after administration of Ang II (10-100 µg/kg) by intra-peritoneal (IP) injection.

Isometric Tension Vasomotor Studies

Vasomotor function was analyzed using isometric tension studies in a wire myograph (Multi-Myograph 610M, Danish Myo Technology, Denmark). Briefly, mice were culled by overdose of inhaled isoflurane. The mesentery was excised from the mouse and placed in cool Krebs-Henseleit buffer (KHB [in mmol·L^{-1}]: NaCl 120, KCl 4.7, $MgSO_4$ 1.2, KH_2PO_4 1.2, $CaCl_2$ 2.5, $NaHCO_3$ 25, glucose 5.5). Segments of second-order artery were carefully dissected free from surrounding fat and connective tissue as described.[4, 5] The mesenteric arteries (2 mm) were mounted on a wire myograph containing 5 ml of KHB at 37°C, gassed with 95% O_2 5% CO_2. After allowing vessels to equilibrate for 30 minutes, the passive tension-internal circumference was determined by stretching to achieve an internal circumference equivalent to 90% of that of a blood vessel under a transmural

pressure of 100 mmHg. The vessel viability was tested using 45 mmol·L^{-1} KCl. Concentration-response contraction curves were established using cumulative half-log concentrations to U46619. Vessels were washed three times with fresh KHB, equilibrated for 20 minutes, and then precontracted to approximately 80% of maximal tension with U46619. Acetylcholine (1 nmol·L^{-1} - 10 µmol·L^{-1}) was used to stimulate endothelium-dependent vasodilatations in increasing cumulative concentrations. Responses were expressed as a percentage of the precontracted tension. Finally, the NO donor sodium nitroprusside (SNP, 0.1 nmol·L^{-1}- 1 µmol·L^{-1}) was used to test endothelium-independent smooth muscle relaxation in the presence of L-NAME.

Determination of Aortic H_2O_2 Production

Aortic H_2O_2 production was determined using the Amplex red (Molecular Probes), as described previously.[4, 6] Briefly, freshly harvested aortic rings (3 x 2 mm) were incubated in 50 µl of KHB containing 2 µM ACh, at 37°C for 60 minutes in the dark, with or without 100 µM L-NAME. After incubation, 50 µl of Amplex Red reagent mixture containing 20 mM Amplex red and 0.4 U/ml horseradish peroxidase was added and buffer fluorescence determined at excitation 530 nm and emission 590 nm. Background fluorescence (without vessels) was subtracted and normalized to aortic dry weight.

Histology and Immunostaining

Second order mesenteric arteries from mice treated with either saline or Ang II (0.4mg/kg/day for 28 days) were harvested following perfusion fixation at 100mmHg. Paraffin-embedded suprarenal aortas and 2nd order mesenteric arteries were stained with Masson-Goldner trichrome and immunohistochemistry for α-smooth muscle actin (Sigma), according to the manufacturer's instructions.

Ultrasound

Abdominal aorta luminal diameter was measured 7 days prior to implantation with an osmotic mini pump and 28 days post-implantation. Mice were anesthetised with isoflurane (1-1.5%) in 100% oxygen and placed on a homoeothermic mat, the abdomen was shaved and luminal diameter of the abdominal aorta measure at 5 points from the renal bifurcation to the diaphragm using a Visualsonics Vevo 2100 with a 22–55 MHz transducer. Body temperature was maintained between 36-37.5°C and heart rate was maintained between 400-500 bpm.

Nitrite and Nitrate Determination

Freshly harvested aortas were preincubated in in Krebs-Henseleit buffer (120 mMn NaCl, 4.7 mM KCl, 1.2 mM $MgSO_4$, 1.2 mM KH_2PO_4, 2.5 mM $CaCl_2$, 25 mM $NaHCO_3$, and 5.5 mM glucose)

for 30 mins at 37°C, then exposed to acetylcholine (1 µM) for 24 hours. Nitrite and nitrate accumulation was measured in samples of buffer using the CLD88 NO analyzer (Ecophysics) and normalized to aortic dry weight.[7]

Western Blot Analysis

Western blots were performed to evaluate protein levels of GTPCH (a gift from S.Gross, Cornell University New York), eNOS (BD Bioscience), iNOS (Abcam), nNOS (Santa Cruz), DHFR (BD Bioscience), in vascular tissues and cells from wild-type and *Gch1*$^{fl/fl}$Tie2cre mice, using standard protocols.

Drugs and Solutions

All drugs were obtained from Sigma-Aldrich (Poole, UK) with the exception of sepiapterin (Schircks Laboratories, Switzerland) and SLIGRL (Abcam, UK). All drugs were dissolved in distilled water, with the exception of indomethacin, which was dissolved in ethanol and U46619 and sepiapterin, which were dissolved in dimethyl sulfoxide (DMSO).

REFERENCES

1. Douglas G, Bendall JK, Crabtree MJ, Tatham AL, Carter EE, Hale AB, Channon KM. Endothelial-specific nox2 overexpression increases vascular superoxide and macrophage recruitment in apoe−/− mice. *Cardiovascular Research*. 2012
2. Crabtree MJ, Tatham AL, Al-Wakeel Y, Warrick N, Hale AB, Cai S, Channon KM, Alp NJ. Quantitative regulation of intracellular endothelial nitric-oxide synthase (enos) coupling by both tetrahydrobiopterin-enos stoichiometry and biopterin redox status: Insights from cells with tet-regulated gtp cyclohydrolase i expression. *J Biol Chem*. 2009;284:1136-1144
3. Fan LM, Douglas G, Bendall JK, McNeill E, Crabtree MJ, Hale AB, Mai A, Li J-M, McAteer MA, Schneider JE, Choudhury RP, Channon KM. Endothelial cell–specific reactive oxygen species production increases susceptibility to aortic dissection. *Circulation*. 2014;129:2661-2672
4. Chuaiphichai S, McNeill E, Douglas G, Crabtree MJ, Bendall JK, Hale AB, Alp NJ, Channon KM. Cell-autonomous role of endothelial gtp cyclohydrolase 1 and tetrahydrobiopterin in blood pressure regulation. *Hypertension*. 2014;64:530-540
5. Chuaiphichai S, Starr A, Nandi M, Channon KM, McNeill E. Endothelial cell tetrahydrobiopterin deficiency attenuates lps-induced vascular dysfunction and hypotension. *Vascul Pharmacol*. 2016;77:69-79
6. Dikalov S, Griendling KK, Harrison DG. Measurement of reactive oxygen species in cardiovascular studies. *Hypertension*. 2007;49:717-727
7. Chuaiphichai S, Crabtree MJ, McNeill E, Hale AB, Trelfa L, Channon KM, Douglas G. A key role for tetrahydrobiopterin-dependent endothelial nos regulation in resistance arteries: Studies in endothelial cell tetrahydrobiopterin-deficient mice. *Br J Pharmacol*. 2017;174:657-671

SUPPLEMENTAL FIGURES AND FIGURE LEGENDS

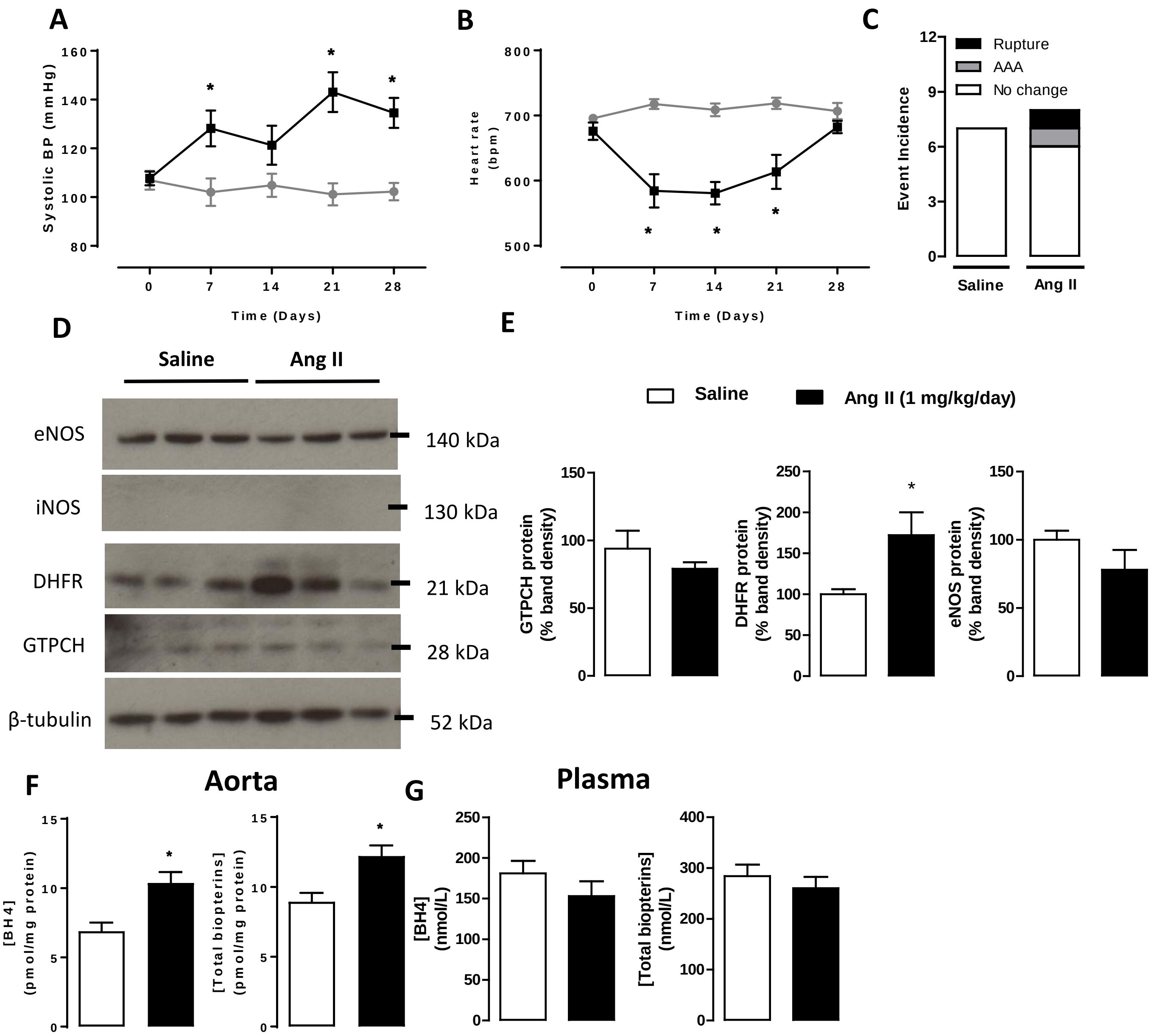

Figure S1. GTPCH and BH4 levels in Ang II-induced vascular dysfunction and hypertension. Osmotic mini-pump containing Ang II (1 mg/kg/day) or saline was implanted in wild-type (C57BL/6) mice. Systolic blood pressure and heart rate measured by non-invasive tail-cuff during 28 days of Ang II infusion. **A,** Systolic blood pressure was significantly increased in Ang II-infused mice after 7 days and was maintained throughout the 28 days infusion. (**P<0.05;* comparing treatment, n=6 to 8 animals per group). **B,** Heart rate significantly decreased in Ang II infused mice following 7, 14 and 21 days of infusion. Heart rate was returned to similar levels as seen in saline infused mice on day 28. **C,** Incidence of AAA and AAA rupture (dead) in Ang II infused mice and saline infused mice. **D,** Representative immunoblots showing eNOS, iNOS, DHFR, GTPCH and β-tubulin (loading control) proteins in wild-type and *Gch1*$^{fl/fl}$Tie2cre mice. **E,** Quantitative data, measured as percentage band density of β-tubulin, showing GTPCH, DHRF and eNOS protein (**P<0.05;* comparing treatment, n=6 to 8 animals per group). **F, and G,** HPLC analysis of biopterins in aortas and plasma respectively from saline-infused mice and Ang II-infused mice. (**P*<0.05 comparing treatment; n=6 animals per group).

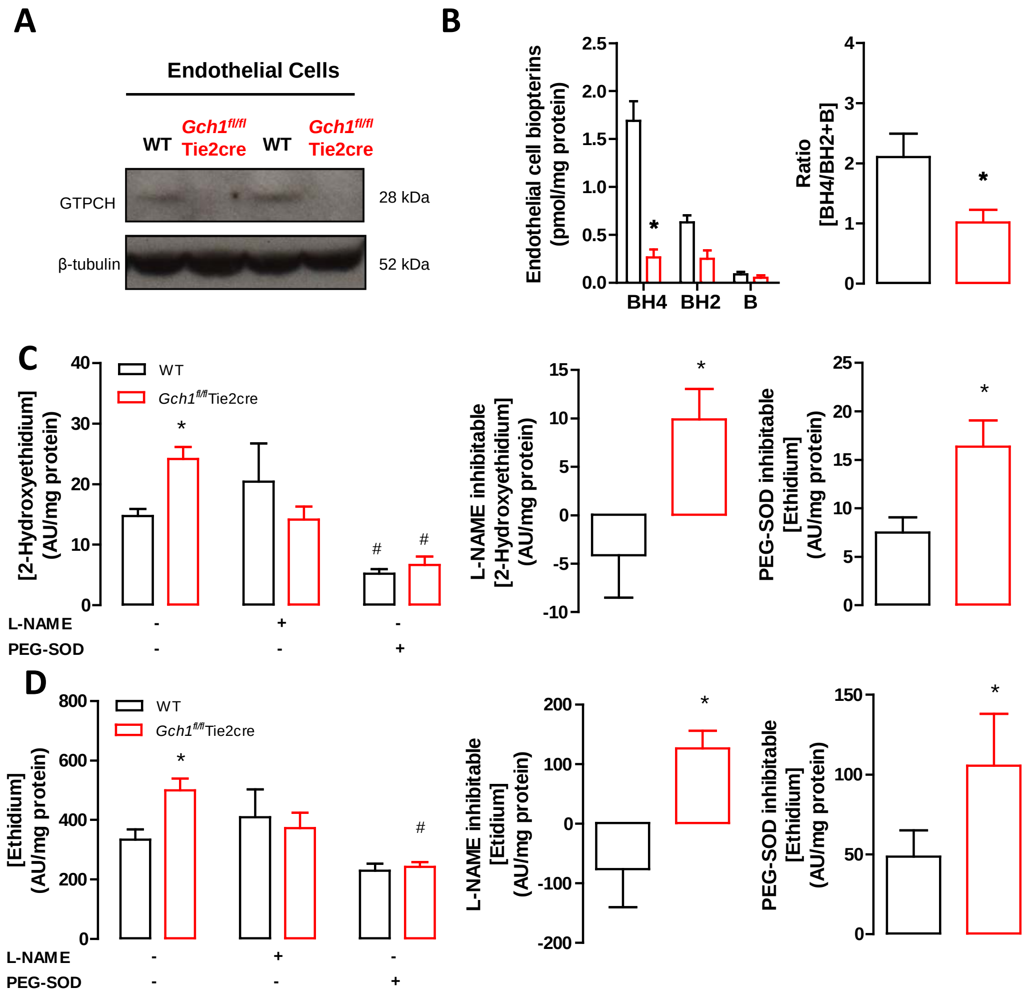

Figure S2. A, Representative immunoblots GTPCH and β-tubulin (loading control) proteins in primary endothelial cells from wild-type and *Gch1*$^{fl/fl}$Tie2cre mice. **B,** BH4, BH2 and biopterin levels, as measured by high-performance liquid chromatography (HPLC), in primary endothelial cells from wild-type and *Gch1*$^{fl/fl}$Tie2cre mice (**P*<0.05; n=5 to 6 animals per group). **C and D**, Superoxide and other reactive oxygen species (ROS) productions detected by dihydroethidium (DHE) HPLC in the presence and absence of either 100 µM L-NAME or 100 units/ml PEG-catalase. Superoxide and other ROS productions as measured by 2-hydroxyethidium and ethidium, respectively, in primary endothelial cells isolated from wild-type and *Gch1*$^{fl/fl}$Tie2cre mice (**P*<0.05 comparing genotype, #*P*<0.05 comparing treatment at baseline; n=6 animals per group).

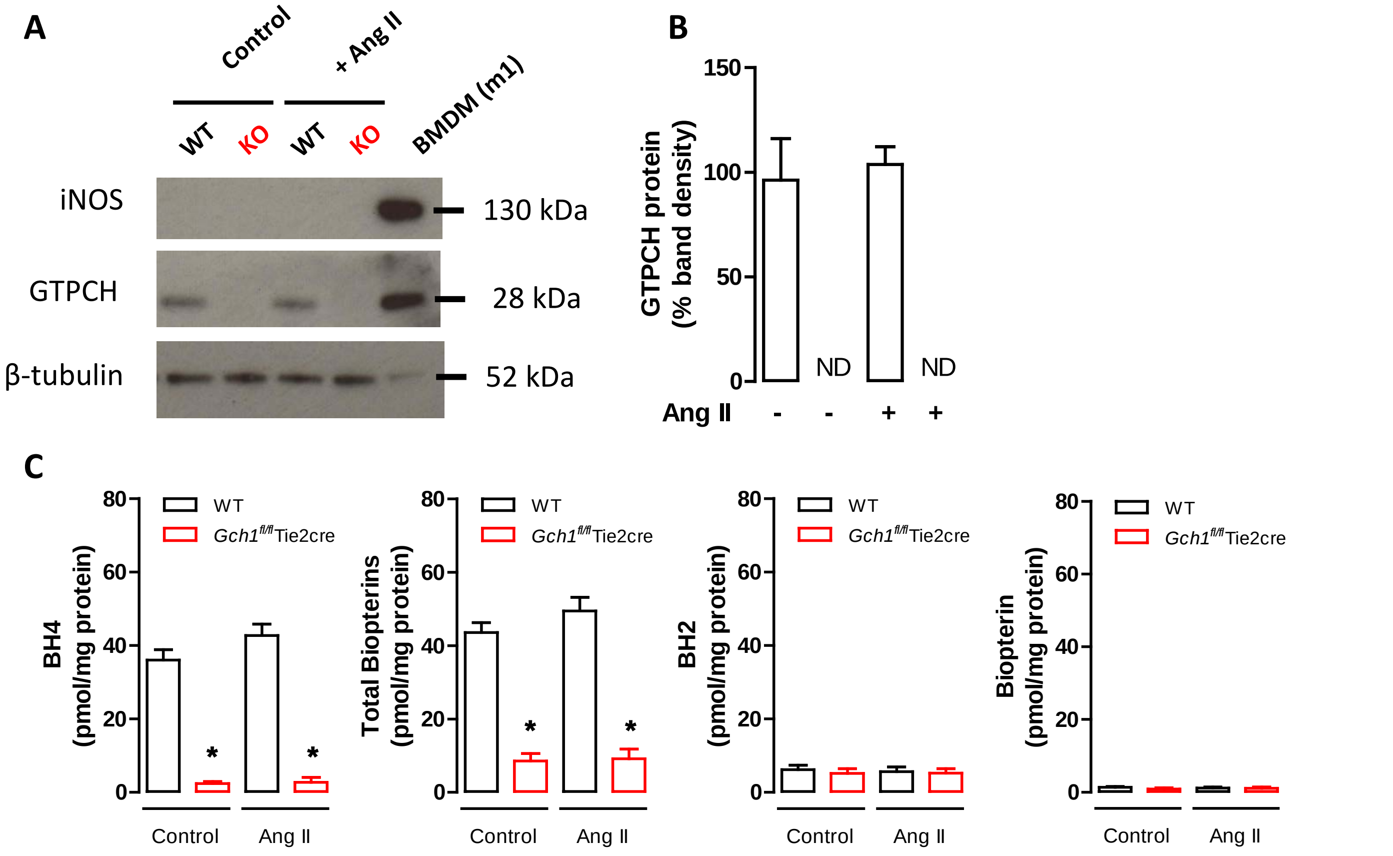

Figure S3. GTPCH and BH4 levels in macrophages from wild-type and *Gch1*$^{fl/fl}$Tie2cre mice with or without Angiotensin II (Ang II) stimulation at 1 µM for 16 hours. **A,** Representative immunoblots showing GTPCH, iNOS, and β-tubulin (loading control) proteins in wild-type and *Gch1*$^{fl/fl}$Tie2cre mice Ang II treated of untreated macrophages. **B,** Quantitative data, measured as percentage band density of β-tubulin, showing GTPCH protein (# *P<0.05*; comparing treatment; n=6 animals per group). **C,** HPLC analysis of biopterins in wild-type and *Gch1*$^{fl/fl}$Tie2cre macrophages treated with or without Ang II (**P<0.05;* comparing genotypes; n=5 to 7 animals per group).
